# Postpartum Women's Experiences of Postnatal Care in Sub‐Saharan Africa: A Qualitative Evidence Synthesis

**DOI:** 10.1111/birt.12872

**Published:** 2024-10-17

**Authors:** Enos Moyo, Perseverance Moyo, Tafadzwa Dzinamarira, Andrew Ross

**Affiliations:** ^1^ School of Nursing & Public Health University of Kwa‐Zulu Natal, College of Health Sciences Durban South Africa; ^2^ Medical Centre Oshakati Oshakati Namibia; ^3^ School of Health Systems and Public Health University of Pretoria Pretoria South Africa

**Keywords:** experiences, postnatal care, postpartum women, qualitative evidence synthesis, sub‐Saharan Africa

## Abstract

**Background:**

Postnatal care (PNC) is a critical service for the health and well‐being of new mothers and newborns. However, in sub‐Saharan Africa (SSA), most efforts to improve maternal and child health have been directed toward enhancing skilled birth attendance and urgent obstetric and neonatal care. This is despite the fact that more than half of maternal deaths globally occur in the postnatal period, with 65% of these occurring in the first week following birth. One of the health system factors influencing PNC utilization is the women's previous PNC experience at healthcare facilities. The aim of this review was to gain a better understanding of women's experiences of PNC in SSA.

**Methods:**

This study followed a qualitative evidence synthesis design. The phenomenon of interest was postpartum women's experiences of PNC in SSA. PubMed, CINAHL, EMBASE, Science Direct, Africa Journals Online (AJOL), SCOPUS, and Google Scholar were searched for peer‐reviewed articles published in English between 2013 and 2023. To assess the quality of the included studies, we used an appraisal tool developed by the Evidence for Policy and Practice Information and Co‐ordinating Centre. Two authors independently extracted relevant data from the included studies. Thomas and Harden's thematic synthesis framework was used to synthesize the data.

**Results:**

Eight articles were used in this review. Seven articles reported on qualitative studies, and one reported on a mixed‐method study. All the included studies fully or partially met the 12 quality assessment criteria. Synthesis of the data resulted in the development of five analytical themes. The five themes were the adequacy of physical examination and communication of the findings, adequacy of PNC information, the quality of interactions with healthcare workers (HCWs), the availability of resources and adequacy of HCWs, and denial of care. The overall confidence in the review's findings was either moderate or high.

**Conclusion:**

Based on our findings, we recommend that countries in the region address staff shortages, implement task shifting, electronic medicine stock management systems, optimal supply chain policies, and train HCWs on PNC and interpersonal communication skills.

## Graphical abstract

This paper critically reviews sub‐Saharan African women’s postnatal care experiences. The review revealed that their experiences are influenced by adequacy of examinations and communication of findings, adequacy of resources and healthcare workers, access to care, and quality of interactions with healthcare workers

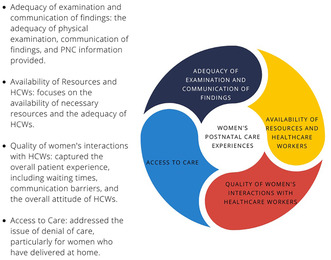

## 1 Introduction

Postnatal care (PNC) is a critical service for the health and well‐being of new mothers and newborns [1]. It is essential for the prevention of maternal and neonatal morbidity and mortality, as well as for the promotion of good health and well‐being. The United Nations (UN) has set targets of reducing the global maternal mortality ratio (MMR) to < 70 maternal deaths per 100,000 live births and the neonatal mortality rate (NMR) to < 12 deaths per 1000 live births by 2030 [2]. Sub‐Saharan Africa (SSA) accounts for 70% of the global maternal deaths in 2020 [3], and the MMR is still above 500 deaths per 100,000 live births [3]. Also worrying is that the NMR for SSA was 27 deaths per 1000 live births in 2020 [4].

In SSA, most maternal and child health efforts have been directed toward enhancing skilled birth attendance and urgent obstetric and neonatal care [5]. More than half of maternal deaths globally occur in the postnatal period, with 65% of these occurring in the first week after birth [6]. Furthermore, about a third of all childhood deaths globally occurs within the first month of life, with 75% occurring in the first week following birth [6]. Preventing maternal deaths requires a multipronged approach. PNC may help prevent these deaths through the promotion of exclusive breastfeeding, the provision of family planning, maternal mental health, and information on nutrition and hygiene [7]. A lack of PNC visits may have several negative repercussions for both the mother and the child. Early breastfeeding cessation, undiagnosed postpartum depression, and anxiety disorders are a few such impacts. Additionally, postpartum women may not receive family planning services if they do not attend PNC follow‐up visits [8]. If PNC services are not utilized, preterm complications, neonatal sepsis, or other neonatal illnesses such as pneumonia, tetanus, and diarrhea may result in neonatal deaths [9].

The utilization of PNC services among postnatal women in SSA is influenced by several factors. These factors can be categorized into individual factors, environmental factors, and healthcare system factors. Some of the individual factors include the marital status of the woman [10], the place of residence [11], the household income [12], the employment status, and the educational level of both the woman and her partner [13]. Environmental factors include the education level of the community [14], community awareness of PNC [15], the influence of peers and the elderly [16], and healthcare utilization of the community [15]. Health system factors included accessibility of healthcare facilities, availability of resources, including staff, availability of community‐based health support, the quality of care at the healthcare facilities, and the previous experience of the women at the healthcare facilities [17].

Understanding women's experience of PNC is important as bad experiences may hinder women from seeking these essential services, leading to maternal and neonatal morbidity and mortality. For the purposes of this review, the postnatal period was defined as the 6 weeks following childbirth. This definition aligns with the World Health Organization's guidelines for essential newborn care and is commonly used in maternal and child health research [7]. This review was conducted to gain a better understanding of women's experience of PNC in SSA.

## 2 Methodology

### 2.1 Study Design

We conducted a qualitative evidence synthesis with thematic synthesis for this study. The study adhered to the Preferred Reporting Items for Systematic Reviews and Meta‐Analysis Protocols (PRISMA‐P) guidelines.

### 2.2 Research Question and Study Inclusion Criteria

The study sought to answer the research question: What are the PNC experiences of postpartum women in SSA? The eligibility criteria for the studies included in this review were guided by the sample, the phenomenon of interest, design, evaluation, and research type (SPIDER) framework. The sample was defined as postpartum women in SSA, the phenomenon of interest as experiences of PNC, and study designs as qualitative and mixed methods. While the research type only included primary research studies, the evaluation of results was concentrated on the postpartum women's subjective experiences. Appendix S1 provides more information on the PubMed search strategy.

### 2.3 Literature Sources and Search Strategy

PubMed, CINAHL, EMBASE, Science Direct, Africa Journals Online (AJOL), SCOPUS, and Google Scholar were searched for peer‐reviewed articles published in English between 2013 and 2023. The search terms we used include “PNC,” “experiences,” “sub‐Saharan Africa,” “postpartum women,” and all countries in SSA. To enhance the sensitivity of our search, the lead author also searched the reference lists of the studies that were included in the review for relevant articles that might have been overlooked during the first search.

### 2.4 Study Selection

All the included articles were exported to the ENDNOTE reference manager [18] and duplicates were removed. The remaining articles were uploaded to Covidence [19]. Two authors (E.M. and P.M.) reviewed the titles and abstracts of the articles independently. The two authors (E.M. and P.M.) also independently screened the articles that were forwarded for full‐text review. Discrepancies were resolved through discussion and consensus between the two authors. However, where this was not achieved, a third author (T.D.) was asked to mediate.

### 2.5 Quality Assessment of Included Studies

We used an appraisal tool developed by the Evidence for Policy and Practice Information and Co‐ordinating Centre for use in a systematic review of healthy eating in children [20] to assess the quality of the included studies. Two authors (E.M. and T.D.) independently assessed all the included studies to determine the extent to which they met the tool's quality appraisal criteria. The criteria for judging the quality of the studies were agreed upon before the commencement of the assessment (Appendix S2).

### 2.6 Data Extraction

Data were extracted from the included studies and entered into a data extraction form that was designed by the authors. The form was initially piloted on two studies that were included in the review and was deemed adequate by the authors. Two authors (E.M. and P.M.) independently extracted relevant data from each study and cross‐checked the findings for accuracy. The information that was extracted includes the first author, the publication year, the country where the study was conducted, the study design, data collection methods, the description of study participants, the data analysis method used, and the findings related to experiences of PNC among postpartum women.

### 2.7 Data Synthesis

We synthesized the retrieved data using Thomas and Harden's thematic synthesis framework. According to Thomas and Harden [21], the framework has three stages namely:

- Line‐by‐line coding: To identify essential concepts and ideas, the extracted material is coded line‐by‐line,
- Development of descriptive themes: The coded data are then grouped together into descriptive themes that capture the key concepts and ideas, and
- Development of analytical themes: The analytical themes are created by further analyzing the descriptive themes to find the underlying linkages and patterns.

Two authors (E.M. and T.D.) initially coded data from two included studies and compared their codes for consistency and congruity. They then went on to code data from all the included studies independently using Microsoft Word's comment function to add codes to the text. Thereafter, the two authors (E.M. and T.D.) developed descriptive themes. The descriptive themes and the related codes, as well as the analytical themes were discussed, reflected upon, and iterated upon by three authors (E.M., P.M., and T.D.) so that they could review and refine them.

### 2.8 Assessment of Confidence in the Study Findings

To evaluate confidence in our findings, we used the Grading of Recommendations Assessment, Development, and Evaluation–Confidence in the Evidence from Reviews of Qualitative Research (GRADE‐CERQual) [22, 23]. The GRADE‐CERQual framework is a systematic approach for assessing the confidence in the findings of qualitative evidence syntheses. We evaluated each finding based on four criteria specified by Lewin et al. [22]. The criteria are coherence, methodological limitations, quality of data, and relevance. Two reviewers (E.M. and T.D.) independently assessed each finding, and the final decision was based on discussion and consensus. Each finding was then assigned a general confidence rating of high, moderate, low, or extremely low.

## 3 Results

**FIGURE 1:**
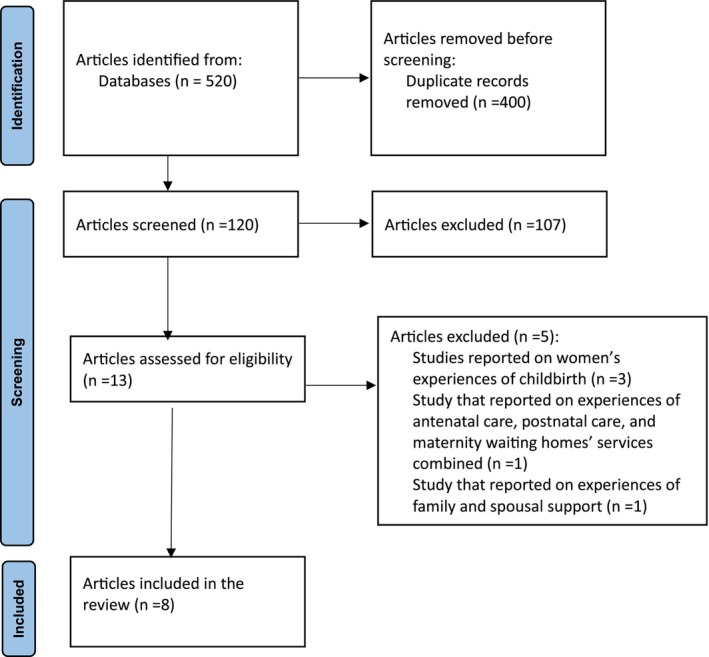
PRISMA flowchart. [Colour figure can be viewed at wileyonlinelibrary.com]

We retrieved 520 articles from all the databases. After removing duplicates, we screened 120 articles. We excluded 107 articles because they were published before 2013, and were quantitative studies, systematic reviews, or meta‐syntheses. Thirteen articles were assessed for eligibility, and we excluded five of them. The remaining eight articles were included in the review, as illustrated in Figure 1. Of the five articles excluded, one was removed for reporting on experiences of family and spousal support [24], another one for reporting on experiences of antenatal care, postnatal care, and maternity waiting homes' services combined [25], and the other three reported on women's experiences of childbirth [26, 27, 28].

### 3.1 Characteristics of Included Studies

Among the eight articles that were included in this review, seven reported on qualitative studies while only one reported on a mixed‐method study [29]. Two articles were from studies conducted in Zambia [30, 31], one reported on a multi–country study that was conducted in Uganda and Zambia [32], one each from South Africa [33], Tanzania [34], Nigeria [35], Namibia [29], and Ethiopia [36]. Three studies used focus group discussions (FGDs) for data collection [31, 32, 35], two used FGDs and in‐depth interviews [29, 36], one each used in‐depth interviews [30] and semi‐structured interviews [33], and another one used FGDs and nonparticipant observations [34]. Five of the included studies [30, 31, 32, 35, 36] used thematic content analysis as a method of data analysis while three [29, 33, 34] used qualitative content analysis. The study participants for all the included studies were postpartum women. More details are presented in Table 1.

**TABLE 1:** Characteristics of included studies.

First author, Publication year	Reference	Country where study was conducted	Study design	Data collection methods	Study participants	Data analysis method
Muleya, 2018	[30]	Zambia	Qualitative Phenomenological study	In‐depth interviews	30 postpartum mothers	Thematic content analysis
Sialubanje, 2023	[31]	Zambia	Cross‐sectional qualitative study	Focus group discussions (FGDs)	80 postpartum women	Thematic content analysis
Sacks, 2016	[32]	Uganda and Zambia	Qualitative descriptive	FGDs	393 women who had delivered recently. 172 were from Uganda and 221 from Zambia	Thematic content analysis
Williams, Brysiewicz, 2017	[33]	South Africa	Qualitative descriptive	Semi‐structured interviews	20 Postpartum women	Qualitative content analysis
Mahiti, 2015	[34]	Tanzania	Qualitative descriptive	FGDs and nonparticipant observation	105 Postpartum women	Qualitative content analysis
Orpin, 2018	[35]	Nigeria	Qualitative phenomenological study	FGDs	32 women who had experienced a normal vaginal delivery	Thematic content analysis
Wesson, 2018	[29]	Namibia	Mixed method	FGDs and in‐depth interviews	Not specified	Qualitative content analysis
Tiruneh, 2021	[36]	Ethiopia	Qualitative phenomenological study	FGDs and in‐depth interviews	12 postpartum women in the FGDs and 3 in in‐depth interviews	Thematic content analysis

### 3.2 Quality Appraisal of Included Studies

All the studies included in this review fully or partially met all the 12 quality assessment criteria. However, all of them only partially met the criterion of actively involving the participants in the design and the conduct of the study. One study [29] partially described the sample and the sampling method. Table 2 shows the results of the quality appraisal process.

**TABLE 2:** Quality appraisal of included studies.

Quality criteria	
**Quality of the study reporting** A = Reported aims and objectives clearly B = The context of the study was adequately described C = The sample and sampling methods were adequately described D = The data collection methods were adequately described E = The data analysis methods were adequately described	**There was good or some attempt to establish the** F = Data collection tools’ reliability G = Data collection tools’ validity H = Data analysis’ reliability I = Data analysis’ validity **Quality of the methods** J = Appropriate data collection methods were used to allow for expression of views K = Appropriate methods were used to ensure that the analysis was grounded in the views L = Participants were actively involved in the design and conduct of the study

First author, Publication year	Reference	Criteria met
Muleya, 2018	[30]	A, B, C, D, E, F, G, H, I, J, K, L^P^
Sialubanje, 2023	[31]	A, B, C, D, E, F, G, H, I, J, K, L^P^
Sacks, 2016	[32]	A, B, C, D, E, F, G, H, I, J, K, L^P^
Williams, 2017	[33]	A, B, C, D, E, F, G, H, I, J, K, L^P^
Mahiti, 2015	[34]	A, B, C, D, E, F, G, H, I, J, K, L^P^
Orpin, 2018	[35]	A, B, C, D, E, F, G, H, I, J, K, L^P^
Wesson, 2018	[29]	A, B, C^P^, D, E, F, G, H, I, J, K, L^P^
Tiruneh, 2021	[36]	A, B, C, D, E, F, G, H, I, J, K, L^P^

Abbreviation: P, partially fulfilled.

### 3.3 Data Synthesis and Review Findings

Synthesis of the data resulted in the development of 11 descriptive themes and five analytical themes. The themes are presented in Table 3 and separately below.

**TABLE 3:** Themes development for postpartum women's experiences of postnatal care.

Contributing articles references	Codes (Summarized for illustrative purposes)	Descriptive themes	Analytical themes
[30, 32]	No physical examination done; physical examination done; Not sure;	Receiving physical examination	Theme 1: Adequacy of physical examination and communication of findings
[30]	Only BP and temperature checked	Inadequate physical examination	
[30]	Results of physical examination not communicated; Results of physical examination communicated;	Communicating findings of physical examination	
[30, 33]	Information on how to take care of baby provided; Information on how to take care of baby provided	Provision of information about baby care	Theme 2: Adequacy of PNC information provided
[30]	Follow‐up dates provided; Follow‐up dates not provided	Provision of information about follow‐up dates	
[29, 30, 31, 32, 33, 34, 35, 36]	Sulky and rude; Helpful; Supportive; Approachable; Discriminating young mothers; Deliberately delaying care.	Good and poor interaction with healthcare providers	Theme 3: Quality of interactions with healthcare workers
[33, 34, 36]	Waiting for long hours to receive PNC services	Duration of waiting period	
[29]	Healthcare workers not speaking the language of the patient	Communication challenges	
[31]	Shortages of essential medicines, supplies, and equipment.	Absence or presence of resources for PNC	Theme 4: Availability of resources and adequacy of healthcare workers
[29, 34]	One nurse providing registration form, vaccinating children, weighing the under‐five babies, and dispensing drugs.	Number of responsibilities for each healthcare worker	
[32]	No service provided due to home delivery	Provision of PNC services according to place of delivery	Theme 5: Denial of Care

#### 3.3.1 Theme 1: Adequacy of Physical Examination and Communication of Findings

This theme was supported by data from two studies [30, 32]. Three descriptive themes that represent this analytical theme are receiving physical examination, inadequate physical examination, and communicating findings of physical examination.

##### 3.3.1.1 Receiving Physical Examination

Some postpartum women reported that they did not receive any physical examination during PNC visits [30]. However, some women felt that they had adequate physical examinations during their PNC visits since the healthcare workers examined them and their babies thoroughly [30].

They examined me and the baby from head to toe particularly the eyes, legs and the vulva [30].

##### 3.3.1.2 Inadequate Physical Examination

A woman in one study reported that even her baby's umbilical cord was not properly tied, and she had to return to the hospital [32]. Other women reported they only had their blood pressure (BP) and temperature checked, but no other examination was performed, even among those who had vaginal tears during birth [30]. However, some women were not sure whether they received adequate physical examination after birth [30].

No, they didn't do that. They haven't examined us yet. They only measured BP and temperature. Am not sure if they examined my private parts. I had a laceration but not sure if they examined it. Even the baby hasn't been examined from head to toe [30].

##### 3.3.1.3 Communicating Findings of Physical Examination

Some women who were examined reported that nothing was explained to them about the findings of the physical examination [30].

They examined me and the baby after delivery, but results were not communicated [30].

However, other women reported that the findings of the physical examinations for themselves and their babies were explained to them [30].

#### 3.3.2 Theme 2: Adequacy of PNC Information Provided

This theme was supported by data from two studies [30, 33]. Two descriptive themes that represent this analytical theme are provision of information about baby care and provision of information about follow‐up dates.

##### 3.3.2.1 Provision of Information About Baby Care

While some women reported that they did not receive enough information about how to take care of their babies, some reported that they received adequate information [30, 33].

I could say they give us enough information, for me it's enough, maybe … I think [33].

##### 3.3.2.2 Provision of Information About Follow‐Up Dates

Some women reported that they did not receive information about follow‐up PNC visits. However, some reported that they did receive adequate information about their follow‐up visits, including vaccinations the baby would receive [30].

mmmmm… yes, I think they are doing a good job. They even explained to me about the postnatal reviews and even the vaccinations that the baby is to receive [30].

#### 3.3.3 Theme 3: Quality of Interactions With HCWs

This theme was supported by data from all the studies included in this review. Some women reported that the healthcare workers were nice, free to interact with, approachable, helpful, and friendly [30, 32]. However, others reported that the HCWs were rude and disrespectful [29, 30, 33, 35], while others reported that they were insulted by HCWs [31, 36]. Some women complained that they waited for long hours to receive PNC services [34]. Although some women attributed this to a shortage of HCWs [34], some felt that it was being done deliberately by the HCWs [33, 36]. Some women complained that they were unable to communicate clearly with the HCWs because the HCWs could not speak their local languages [29].

When I went to the clinic, we found the nurse who was very helpful and friendly. She injected my baby, gave me vitamin A, and advised me to exclusively breastfeed my baby for the first 6 months [32].

Services are not good, as you might arrive early in the morning, say at nine o'clock, and receive services very late [34].

#### 3.3.4 Theme 4: Availability of Resources and Adequacy of Healthcare Workers

This theme was supported by data from three studies [29, 31, 34]. The women reported that there were shortages of essential medicines, supplies, and equipment, which prevented them from receiving some PNC services [31]. Women complained that one nurse had several responsibilities, which made it difficult for them to provide PNC quickly.

You might find that you have one nurse stamping the registration form, vaccinating children, weighing the under‐five babies, and dispensing drugs [34].

#### 3.3.5 Theme 5: Denial of Care

Only one study provided data to support this theme [32]. Some women complained that they were not attended to at the healthcare facilities and were not given health cards for their babies because they had delivered at home. Some women even reported that when they took their sick babies to the healthcare facilities, the HCWs said that there were no medicines available just because they had delivered at home [32].

Nurses at the hospital refuse to give under‐five cards and send us back home and tell us to come back when we have reasons for delivering at home. So, we are punished for that and told we did it (delivered at home) willingly [32].

### 3.4 Confidence in the Review's Findings

All the findings of this review were subjected to GRADE‐CERQual confidence assessments. The results of the assessments are presented in Table 4. The overall confidence in the review's findings was either moderate (*n* = 7 findings) or high (*n* = 4 findings). The downgrading of the confidence was related to the adequacy of data since the findings with moderate confidence were just from a single study. The authors agreed that there were no coherence concerns on the five findings that were supported by more than one study. However, six of the findings were not judged on coherence since they were each supported by one study. The authors also agreed that there were minor concerns about the methodology of all the studies emanating from the fact that participants were not actively involved in the design of the studies. Furthermore, the authors agreed that there were no concerns about the relevance of all the studies in the review since all the studies sought to investigate postpartum women's experiences of PNC, which was the research question for the review.

**TABLE 4:** GRADE‐CERQual summary results.

Findings	Contributing articles references	Methodological limitations	Coherence	Adequacy	Relevance	Overall confidence
Analytical Theme 1: Adequacy of physical examination and communication of findings						
No physical examination done; physical examination done; not sure;	[30, 32]	Minor concerns	No concerns	No concerns	No concerns	High
Only BP and temperature checked	[30]	Minor concerns	N/A	Minor concerns	No concerns	Moderate
Results of physical examination not communicated; Results of physical examination communicated;	[30]	Minor concerns	N/A	Minor concerns	No concerns	Moderate
Analytical Theme 2: Adequacy of PNC information provided						
Information on how to take care of baby provided; Information on how to take care of baby provided	[30, 33]	Minor concerns	No concerns	No concerns	No concerns	High
Follow‐up dates provided; Follow‐up dates not provided	[30]	Minor concerns	N/A	Minor concerns	No concerns	Moderate
Analytical Theme 3: Quality of interactions with healthcare workers						
Sulky and rude; Helpful; supportive; Approachable; Discriminating young mothers; deliberately delaying care	[29, 30, 31, 32, 33, 34, 35, 36]	Minor concerns	No concerns	No concerns	No concerns	High
Waiting for long hours to receive PNC services	[33, 34, 36]	Minor concerns	No concerns	No concerns	No concerns	High
Healthcare worker not speaking the language of the patient	[29]	Minor concerns	N/A	Minor concerns	No concerns	Moderate
Analytical Theme 4: Availability of resources and adequacy of healthcare workers						
Stock‐outs of essential medicines, supplies, and equipment.	[31]	Minor concerns	N/A	Minor concerns	No concerns	Moderate
One nurse providing registration form, vaccinating children, weighing the under‐five babies, and dispensing drugs.	[29, 34]	Minor concerns	No concerns	Minor concerns	No concerns	Moderate
Analytical Theme 5: Denial of care						
No service provided due to home delivery	[32]	Minor concerns	N/A	Minor concerns	No concerns	Moderate

Abbreviation: N/A, not applicable.

## 4 Discussion

This review examines how postpartum women in SSA experience PNC for themselves and their babies. Although the contexts of the studies differed, the narratives were similar in content. For example, all the studies reported both "good" and "bad" attitudes of HCWs. Several factors have been identified as contributing to negative or disrespectful attitudes of HCWs toward postpartum women. These include staff shortages, a lack of equipment and supplies, a lack of managerial support, work overload, and the attitude of patients toward the HCWs [37]. Bad attitudes of HCWs toward women have been associated with delays in seeking healthcare and seeking medical care from untrained providers such as traditional healers [38]. The findings of this review concur with those of a Canadian study which revealed that some women received inadequate physical examination or no physical examination at all during their PNC visits [39]. This may be attributed to the shortage of staff at the healthcare facilities and/or to a lack of knowledge regarding the appropriate examination needed during PNC visits by HCWs. The shortage of staff may also have contributed to the long hours of waiting women endured.

The finding of this review revealed that some postpartum women did not receive adequate PNC information from HCWs concurs with that of a Tanzania study [40]. In the Tanzanian study, only about 39% of HCWs were observed discussing any of the eight topics covered during PNC. In addition, HCWs at hospitals or health centers were more likely to provide adequate PNC information compared to those at dispensaries [40]. A study conducted in Sweden also revealed that women did not receive adequate information on some aspects of PNC [41]. HCWs may fail to provide adequate information in SSA due to several reasons such as work overload, understaffing, a lack of training on PNC, and a lack of resources such as teaching materials [42].

Findings from this review also align with a study conducted in low‐ and lower‐middle‐income countries that found shortages of medicines and supplies for PNC services at healthcare facilities as barriers to maternal and child health services utilization [43]. The shortage of medicines and supplies can be attributed to a lack of pharmacists and pharmacy assistants at healthcare facilities who are trained in the procurement of resources. In addition, the use of manual medicine stock management systems may also contribute to shortages [44]. Our finding that some women were denied PNC services because they had not given birth at healthcare facilities is similar to that of a systematic review conducted by Lythgoe et al. [45]. Some HCWs use denial of care as a punishment for giving birth at home [46]. However, such a practice may prevent women from accessing PNC out of fear that they will be humiliated. The language barriers between patients and HCWs identified in this review were reported in one study among South Asian women [47]. Language barriers are more common in multilingual societies, and this may lead to miscommunication and discomfort between women and HCWs.

To address the shortage of HCWs in SSA, we recommend increasing training and offering competitive compensation packages [48]. Task shifting can help alleviate workload and ensure adequate PNC services [49]. Electronic medicine stock management systems can help prevent supply shortages [50]. Training HCWs in PNC and interpersonal communication skills can improve their knowledge and attitudes toward postpartum women, ultimately enhancing the quality of care provided [51, 52].

One of the strengths of this review was that we used a framework and a clear search strategy to select the relevant studies, making the review replicable. Additionally, two authors were independently involved in the selection and screening of the results, as well as data extraction and synthesis. The generated codes and themes were agreed upon by all the authors, and this enhanced the trustworthiness of the findings. The trustworthiness of the findings was also enhanced by the quality appraisal of the included studies and an assessment of the confidence in the review findings. However, this review also had some limitations. One of the limitations is that only seven databases were searched, making it possible that some articles could have been missed. The other limitation is that only articles published in English were retrieved, and this might have introduced language bias. Finally, one of the themes was only developed from data derived from a single study, making it impossible to determine the coherence of that theme.

## 5 Conclusion

This review identified five key themes related to women's experiences of PNC in SSA. To improve PNC experiences, interventions must address specific needs and barriers, including staff shortages, resource availability, and healthcare provider training.

## Conflicts of Interest

The authors declare no conflicts of interest.

## Supporting information


Appendix S1‐S2

## Data Availability

The data that support the findings of this study are available from the corresponding author upon reasonable request.
