# Supplementary material for: Postpartum Women's Experiences of Postnatal Care in Sub‐Saharan Africa: A Qualitative Evidence Synthesis
Source: Birth. 2024 Oct 17;52(1):5–15. doi: 10.1111/birt.12872 (PMC11829267; doi:10.1111/birt.12872)
Supplement: Supplementary file 1 — Appendix S1‐S2 [file BIRT-52-5-s001.docx]

**Appendix S1: PubMed Full Search Strategy**

| Date | 26.08.2023 |
| --- | --- |
| Search Strategy | (("postnatal care"[MeSH Terms] OR ("postnatal"[All Fields] AND "care"[All Fields] AND "experiences"[All Fields]) OR "postnatal care experiences"[All Fields]) AND ("postpartum women"[Subheading] OR ("postpartum"[All Fields] AND "women"[All Fields] AND ("africa south of the sahara"[MeSH Terms] OR ("africa"[All Fields] AND "south"[All Fields] AND "sahara"[All Fields]) OR "africa south of the sahara"[All Fields] OR ("sub"[All Fields] AND "saharan"[All Fields] AND "africa"[All Fields]) OR "Saharan africa"[All Fields]) AND "nigeria"[All Fields] OR "uganda"[All Fields] OR "south africa"[All Fields] OR "cameroon"[All Fields] OR "ghana"[All Fields] OR "sudan"[All Fields] OR "mozambique"[All Fields] OR "senegal"[All Fields] OR "kenya"[All Fields] OR "malawi"[All Fields] OR "tanzania"[All Fields] OR "zimbabwe"[All Fields] OR "zambia"[All Fields] OR "togo"[All Fields] OR "burundi"[All Fields] OR "mauritius"[All Fields] OR "angola"[All Fields] OR "mali"[All Fields] OR "ethiopia"[All Fields] OR "rwanda"[All Fields] OR "sierra leone"[All Fields] OR "liberia"[All Fields] OR "chad"[All Fields] OR "niger"[All Fields] OR "namibia"[All Fields] OR "cote d’ivoire"[All Fields] OR "south sudan"[All Fields] OR "botswana"[All Fields] OR "democratic republic of congo"[All Fields] OR "somalia"[All Fields] OR "benin"[All Fields] OR "burkina faso"[All Fields] OR "zambia"[All Fields] OR "equatorial guinea"[All Fields] OR "gabon"[All Fields] OR "guinea"[All Fields] OR "guinea-bissau"[All Fields] OR "lesotho"[All Fields] OR "eswatini"[All Fields] OR "the gambia"[All Fields] OR "eritrea"[All Fields] OR "madagascar"[All Fields] OR "mauritania"[All Fields] OR "seychelles"[All Fields] OR "central african republic"[All Fields] AND ("2013/01/01"[PubDate] : "2023/08/26"[PubDate]) |
| Number retrieved | 45 |

**Appendix S2: Minimum criteria for quality assessment (adapted from Thomas *et al.* 2003)**

| ***Quality Criteria*** | | ***Comments (if useful)*** |
| --- | --- | --- |
| ***Quality of study reporting*** | A - Aims and objectives clearly reported | Y = Clear statement of aim is provided |
|  | B - Adequately described the context of the research | Y = Adequate background to the study provided |
|  | C - Adequately described the sample & sampling methods | Y = At a minimum the report must include demographics on numbers of postpartum women, and age of participants (either range or mean).  P = Some, but not all of the above  N = No information other than numbers taking part  Sampling methods  Y = Broadly described but explicitly stated (e.g., purposive, convenience, snowball, etc).  P = Some detail, but description of specific broad approach not provided  N = No information at all provided as to sampling approach |
|  | D - Adequately described the data collection methods | Y = explicitly described |
|  | E - Adequately described the data analysis methods | Y = explicitly described |
| ***There was good or some attempt to establish the*** | F - Reliability of the data collection tools | Qualitative study: Y for use of interview guide and what informed this. |
|  | G - Validity of the data collection tools | As above |
|  | H - Reliability of the data analysis | Refers to analysis of qualitative data only.  Y = A valid (e.g., thematic analysis, etc.,) approach described  P = Some details and appears like a valid approach but not explicit  N = Appears formal/structured but explicit details not provided |
|  | I - Validity of the data analysis | As above |
| ***Quality of the methods*** | J - Used the appropriate data collection methods to allow for expression of views | Y = For qualitative study (unless questions are very structured might be P or N) |
|  | K - Used the appropriate methods for ensuring the analysis was grounded in the views | Linked to H and I above – if valid and reliable data analysis this will be Y |
|  | L - Actively involved the participants in the design and conduct of the study | Y = Clear statement of PPI involvement from the outset (informing design/protocol development, etc.) |

*Y: Yes, N: No, P: Partially
